# Supplementary material for: Early full-day leg movement kinematics and swaddling patterns in infants in rural Guatemala: A pilot study
Source: PLoS One. 2024 Feb 29;19(2):e0298652. doi: 10.1371/journal.pone.0298652 (PMC10903813; doi:10.1371/journal.pone.0298652)
Supplement: Attachment — Submitted filename: Rebuttal_Letter.docx [file pone.0298652.s001.docx]

Rebuttal letter

The authors would like to thank the reviewers for their fair reviews and detailed list of constructive suggestions. We greatly appreciate the opportunity to submit a minor revision of our paper for your renewed consideration. Below we specify how we met journal requirements and respond to reviewers’ comments and point to the corresponding changes we made in the paper.

Journal requirements

1. Please ensure that your manuscript meets PLOS ONE’s style requirements, including those for file naming.

- We have reviewed the style requirements and updated the manuscript. We have also regenerated figures and saved them as ‘Fig1.tiff’, ‘Fig2.tiff’ and etc.

2. In the ethics statement in the Methods, you have specified that verbal consent was obtained. Please provide additional details regarding how this consent was documented and witnessed, and state whether this was approved by the IRB.

- Verbal consent was approved by the IRB. Further details are now provided in the methods of the paper.

3. Please include a complete copy of PLOS’ questionnaire on inclusivity in global research in your revised manuscript.

- Please see attached questionnaire.

4. Note on depositing data.

- De-identified data will be deposited in the NIH DASH repository once all analyses are completed.

5. Request for the relevant accession numbers or DOIs necessary to access your data.

- As noted above, deidentified data will be deposited in a public repository once all analyses are completed, but these are still ongoing. Therefore, we have changed these details for the time being to: “data is available from the corresponding authors upon reasonable request.”

6. Figure 1 contains copyrighted images.

- A new Fig 1 is prepared. As for the image of the sensor used in the study, we took picture of the sensor we purchased. We inquired the manufacturer (APDM WEARABLE TECHNOLOGIES INC.) about using an image of the sensor and received a response from its legal department that any publicly available image does not need its permission including images on the website. I attach the email communication that confirms the use of the image (APDM_Opal_sensor_image_use_permission.pdf)

7. Please review your reference list to ensure that it is complete and correct.

- Reference 1 has erratum but was not retracted. The reference is most up to date with the correction made (on 9/17/2018). The correction below was already reflected in the reference we included:

*The following sentence should have been included in the Acknowledgments section of this Article: “We acknowledge the contribution of Charles R J Newton (University of Oxford, Oxford, UK) to an earlier version of this article.”*

Reviewer 1’s concerns:

1. Title needs to be modified. “Pilot Study” should appear in the title.

- The revised title now indicates that this is a pilot study.

2. Abstract: trial design is not mentioned

- The revised abstract (Methods) now clarifies that we conducted a longitudinal observational study.

3. Method: How was sample size determined?

- As stated in the manuscript, “we decided a priori that a sample of 40 infants would be sufficient for testing for associations and determining effect sizes for future studies based on a recently completed clinical trial assessing infants across 6 months at Maya Health Alliance [16] .” In previous work, we found large effect sizes (Cohen’s d = 0.8 – 1.3) for differences in leg movement quantity and acceleration between infants with typical development and infants broadly at risk for developmental delay. Here, we used G*Power to determine that with a sample size of 30 infants we would be able to detect a difference across 6 months between children of one-third to one-half of SD with 80% power, alpha = 0.05 and intra-individual correlations of 0.5-0.8.

4. Discussion: The discussion section needs to be described scientifically. Kindly frame it along the following lines:
   a. Main findings of the present study
   b. Comparison with other studies
   c. Implication and explanation of findings
   d. Strengths

- a. The Discussion section now does not have sub-headings. Originally the first two subheadings were the main findings of the study. Instead, it is now more explicitly stated as below:
  “The first main finding of the present study is that …” (3^rd^ sentence of the first paragraph)
  “The second main finding of this study is that …” (1^st^ sentence of the second paragraph)
  b. In the first paragraph, a new citation [17] was inserted to further illustrate one interpretation of the result. In the second paragraph, comparisons were already made (citations 20, 21 and 22).
  c. Implication and explanation of finding were also provided in the original manuscript at the following positions:
  - 4^th^ sentence of the first paragraph
  - last half of the second paragraph
  d. Strengths and limitations are now summarized after the Discussion section.

Reviewer 2’s concerns: NA

Reviewer 3’s concerns:

1. Please add a paragraph in the introduction section to indicate the term “swaddling”.

- The last paragraph of the **Introduction** lists swaddling as one covariate. We added an additional sentence in the last paragraph better defining swaddling: Swaddling is the common practice in rural Guatemala and many other settings of keeping the infant tightly wrapped in fabric while sleeping and also, frequently, when carried on the caregiver’s back when performing chores or when out of the house.

2. You should write about the validity and reliability of the used sensors.

- We have added this sentence (with the relevant citations) at the end of the *Apparatus* paragraph in **Methods**: “The validity of measuring the quantity, duration, and acceleration of infant leg movements has been reported previously.”

3. Please, write more details about precautions while wearing the sensors and how they are stabilized.

- We have inserted this sentence to the *Apparatus* paragraph in **Methods**: “The size of the pocket was designed specifically to hold the sensors in place, and a variety of sizes of legwarmers to choose from provided a close but not too tight fit for each infant.”

4. Why you depend upon evaluating the movement of lower limb and neglected the movement of upper limbs. If you didn’t have an explanation, please write that in limitation of the study.

- There is no reason to expect significant differences in overall movement quantity or characteristics of lower limbs compared to upper limbs in these infants. As wearing 4 sensors would be unnecessarily burdensome for the participants and require many more resources, we opted to focus on quantifying the movement of lower limbs as a representation of neuromotor control. Lower limb movement in early life has been reported to be associated with later walking onset of infants, making it one reasonable measure for the neurodevelopmental status of the infants.

5. Also, the nature of nutrition and its variance between infants should be mentioned in the limitation of the study.

- We have added the following sentence to the Limitations paragraph: Individual level variability among infants in key covariables, such as nutritional status, may have obscured the magnitude of some findings in this small sample size; we mitigated this by using linear mixed models includes random effects for individuals and fixed effects for key covariables.

6. You mentioned that “infants with medical illness were excluded from the study, please give examples.

- We have added a phrase (*Participants and Recruitment Procedures* paragraph in **Methods**) to elaborate: “including heart disease, kidney disease, congenital abnormality, genomic syndromes and severe neurological deficits.”
